# Determination of elevated eosinophil to lymphocyte ratio, eosinophil to neutrophil ratio, eosinophil to monocyte ratio and its association with severe vitiligo: A retrospective cohort study

**DOI:** 10.1371/journal.pone.0296626

**Published:** 2024-02-12

**Authors:** Sarah Weissmann, Nitzan Burrack, Inbal Golan-Tripto, Amir Horev

**Affiliations:** 1 Faculty of Health Sciences, Ben-Gurion University of the Negev, Beer Sheva, Israel; 2 Clinical Research Unit, Soroka University Medical Center, Beer Sheva, Israel; 3 Pediatric Pulmonary Unit, Soroka University Medical Center, Beer Sheva, Israel; 4 Pediatric Dermatology Service, Soroka University Medical Center, Beer Sheva, Israel; Universiti Malaysia Sabah, MALAYSIA

## Abstract

**Background:**

Hematological markers such as eosinophil-to-lymphocyte (ELR), eosinophil-to-neutrophil (ENR), and eosinophil-to-monocyte (EMR) ratios may be used in the assessment of skin diseases. However, the relationship with vitiligo remains unclear.

**Objectives:**

We investigated ELR, ENR, and EMR in vitiligo patients and the association with severe vitiligo.

**Methods:**

This study included patients of all ages diagnosed with vitiligo between 2005–2020. ELR, EMR, and ENR were calculated from complete blood counts within 30 days of the first recorded vitiligo diagnosis and 12–18 months before the vitiligo diagnosis. We evaluated the associations between ELR, ENR, and EMR and vitiligo.

**Results:**

9,826 vitiligo patients (8,398 adults and 1,428 children) and 8,951 age and sex-matched controls were included in this study. We found that ELR, EMR, and ENR were higher in children than in adults. Patients with vitiligo had higher ENR and EMR than their matched controls. Patients with severe vitiligo had higher ELR, ENR, and EMR compared to patients with mild vitiligo. Patients with increased ENR and EMR 12–18 months before the vitiligo diagnosis had a higher chance of having severe vitiligo (ENR: OR = 7.40, p<0.001, EMR: OR = 3.17, p<0.001).

**Conclusion:**

ELR, ENR, and EMR may be used in the assessment and prognosis of vitiligo, especially in severe cases.

## Introduction

Vitiligo is a skin disorder caused by idiopathic melanocyte destruction and characterized by the development of areas of depigmented skin lesions. Vitiligo has a varying prevalence worldwide, ranging from 1% in the United States and Europe to greater than 8% in other regions [1]. While the pathogenesis is not fully understood, there are a few theories regarding the cause of melanocytes’ death. It has been recently proposed that melanocytes in vitiligo patients have intrinsic defects that make them more susceptible to the oxidative stress, to which epidermal cells are typically exposed [2]. In combination with death due to increased stress, studies have also proposed that there is a dysregulated innate immune system response to oxidative stress. Vitiligo has also been thought to be caused by melanocyte-specific cytotoxic CD8+ T cell destruction, and though the absolute number of lymphocytes remains stable in vitiligo patients, the proportion of CD8+ T cells to CD4+ T cells is frequently increased [3]. Some studies have suggested that Th17 cells which produce IL-17A and IL-17F interact with eosinophils, triggering a Th2 response in vitiligo [4]. The immune-mediated destruction of melanocytes seen in vitiligo has been linked to co-occurrence of other autoimmune conditions such as thyroid diseases, rheumatoid arthritis, diabetes mellitus, and alopecia areata, possibly indicating a greater systemic inflammatory and autoimmune state [1].

Hematological ratios have been recently used in the assessment and prognosis of inflammatory diseases. Common ratios include the eosinophil to lymphocyte ratio (ELR), eosinophil to monocyte ratio (EMR), and eosinophil to neutrophil ratio (ENR) and have been associated with inflammatory and atopic skin conditions such as atopic dermatitis, psoriasis vulgaris, lupus erythematosus, vitiligo, rosacea, erythema nodosum, urticaria, and dermatomyositis [5]. Eosinophils are known for their cytotoxic function and ability to cause severe tissue damage through the release of granules and other cytotoxic proteins [6]. Eosinophils have been associated with several autoimmune diseases and perpetuate immune system dysfunction [6]. Due to the proposed systemic and autoimmune inflammatory pathophysiology of vitiligo, we hypothesized that ELR, EMR, and ENR will be elevated in patients with vitiligo and can be used as a predictor for disease severity.

The primary objective of this study was to evaluate the values of ELR, EMR, and ENR among patients with vitiligo. In addition, we aimed to compare these ratios between children and adults with vitiligo, patients with mild and severe vitiligo, and patients with their healthy matched controls.

## Materials and methods

### Study design

We performed a deidentified retrospective, population-based, cohort study between 2005 and 2020 on patients and controls of all ages diagnosed with vitiligo, insured within the southern district of Clalit HMO (Health Maintenance Organization) or seen at Soroka University Medical Center (SUMC). Clalit HMO is the largest public healthcare provider organization in Israel and serves more than half of Israel’s population, covering around 4,600,000 people (and over 750,000 people in Southern Israel). SUMC is a 1,200-bed tertiary hospital covering a large geographic area in the south of Israel. The study was approved by the local Ethics Committee of SUMC and has been granted an exemption from requiring informed written consent according to the Ethics Committee of SUMC, approval number [0434-15-SOR].

### Study population

We included all children (ages 0–18 years) and adults diagnosed with vitiligo (using ICD-10 codes found in Appendix 1) who were insured by Clalit HMO or visited SUMC (n = 9,826). We obtained demographic and medical information recorded by the primary care physicians, dermatologists and/or the Admission-Discharge-Transfer (ATD) hospital system of SUMC. Data were extracted using a Clalit’s Data sharing platform powered by MDClone software on September 22, 2022. MDClone is a big-data platform with data-synthesizing capabilities. The diagnoses of vitiligo were made by the patient’s primary physician or by a dermatologist. We included all patients diagnosed with vitiligo and excluded patients with acute or chronic infections, malignancies, or a history of surgery within 30 days of their blood test. Patients without a blood test 12–18 months before diagnosis of vitiligo were also excluded. Controls were matched by age and sex, and nearest blood count to their matched patient was taken. Controls with acute or chronic infections, malignancies, or a history of surgery within 30 days of the blood test were excluded as well.

### Blood count parameters

ELR, EMR, and ENR were calculated from complete blood counts within 30 days of the first recorded vitiligo diagnosis and 12–18 months before the vitiligo diagnosis for a baseline comparison. Patients with no blood tests within 30 days of their vitiligo diagnosis and 12–18 months before their diagnosis were excluded.

Patients were classified as having either a mild or severe case of vitiligo. Patients were considered to have severe vitiligo if they were prescribed at least one systemic vitiligo-related medication (corticosteroids, mycophenolate mofetil, baricitinib, tacrolimus, cyclosporine, upadacitinib, or tofacitinib). All other patients were considered to have mild vitiligo.

MDClone output included a baseline event (vitiligo diagnosis), recorded age, gender, socioeconomic status (SES), ethnicity, and insurance. In addition, neutrophil count, lymphocyte count, eosinophil count, platelet count, and monocyte count were recorded from laboratory results at the time of vitiligo diagnosis as well as 12–18 months before that diagnosis. The socioeconomic score was determined by the Central Bureau of Statistics according to the zip code of the patient. The blood eosinophil/lymphocyte ratio (ELR), eosinophil/monocyte ratio (EMR), and eosinophil/neutrophil ratio (ENR) was also calculated by dividing the eosinophil count by the lymphocyte, monocyte, and neutrophil count, respectively. Patients were divided into two groups according to severity, as defined above.

### Statistical analysis

Continuous variables were compared using the Student’s t-test or the Mann-Whitney U test. Categorical variables were compared using the Chi-squared test or Fisher’s exact test. The measure of association between variables was estimated with Spearman’s rho test, and a multivariable logistic regression was subsequently performed. We used 95% confidence intervals (95% CI), and a P-value < 0.05 was considered statistically significant. The receiver operating characteristic (ROC) curve was used to assess the predictive value of hematologic ratios on severity. We obtained optimal cut-off values using the Youden’s Index. All statistical analyses were done by using R software (version 4.0.2).

## Results

**Fig 1:**
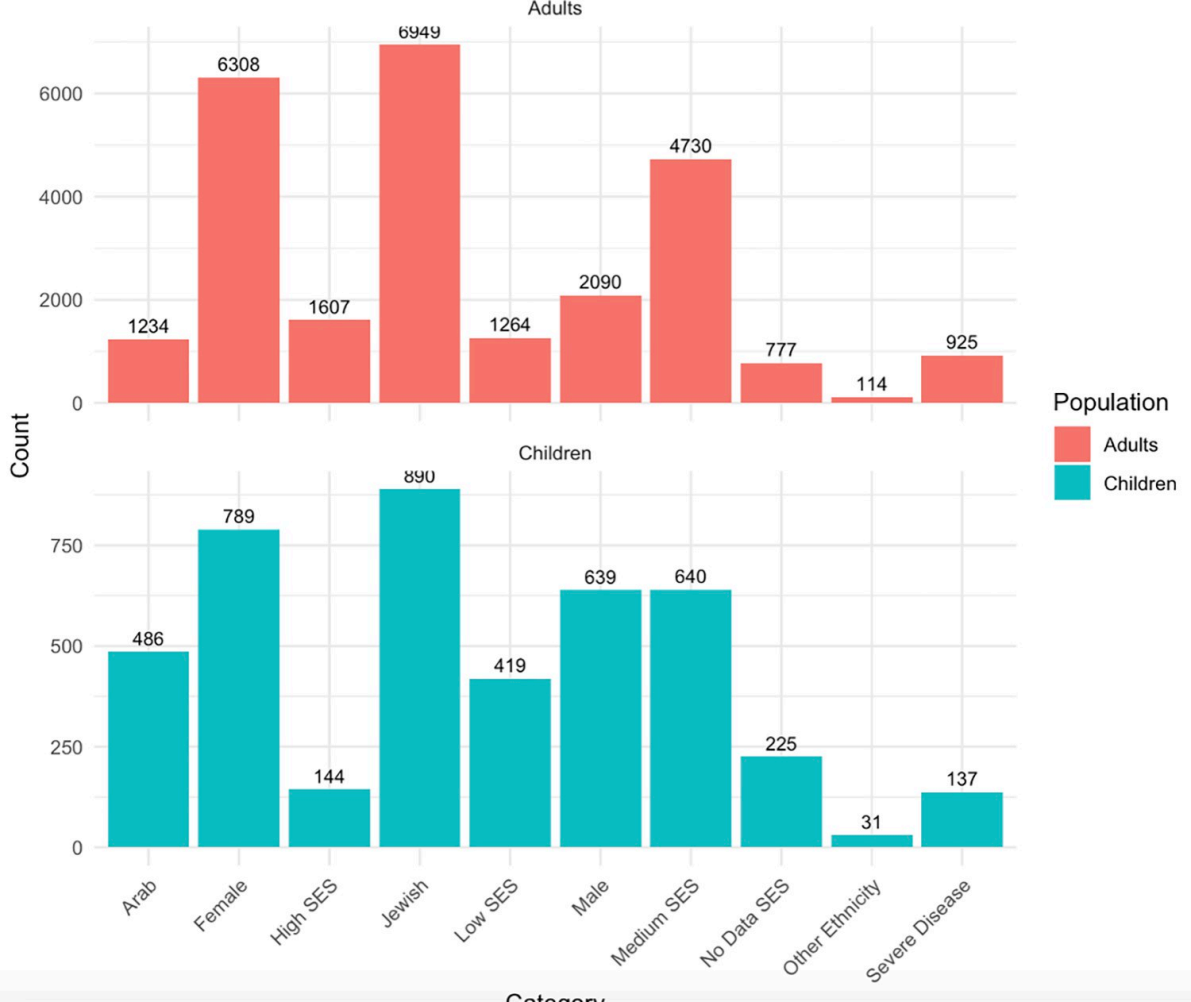
Demographics of children vs adults.

A total of 9,826 patients with vitiligo were included in this study: 8,398 adults and 1,428 children. Comparisons of demographic characteristics and hematologic ratios of the adults and children with vitiligo are summarized in Table 1 and Fig 1. The mean age of adults was 45.61±18.38 years, and the mean age of children was 9.36±5.75 years. There was a significant difference in the gender of children and adults with vitiligo; 2,090 (24.9%) of the adults were male, while 639 (44.7%) of the children were male. A higher percentage of children were Arab compared to adults (34.0% vs 14.7%, p<0.001). In addition, a higher percentage of children with vitiligo were classified as having a lower socioeconomic score than adults with vitiligo (29.3% vs 15.3%, p<0.001).

**Table 1:** Clinical and demographic characteristics of patients with chronic vitligo, stratified by age group.

	Adults	Children	p-value
n	8398	1428	
**Gender (%)**			
**Male**	**2090 (24.9)**	**639 (44.7)**	**<0.001**
**Female**	**6308 (75.1)**	**789 (55.3)**	
**Socioeconomic score (%)**			**<0.001**
**High**	**1607 (19.1)**	**144 (10.1)**	
**Medium**	**4730 (56.3)**	**640 (44.8)**	
**Low**	**1264 (15.3)**	**419 (29.3)**	
**No Data**	**777 (9.3)**	**225 (15.8)**	
**Ethnicity (%)**			**<0.001**
**Arab**	**1234 (14.7)**	**486 (34.0)**	
**Jewish**	**6949 (82.7)**	**890 (62.3)**	
**Other**	**114 (1.4)**	**31 (2.2)**	
Severity (%)			0.121
Severe	925 (11.0)	137 (9.6)	

SD: Standard Deviation

P-value < 0.05 was considered statistically significant

*Chi square test was used for all variables

We found that ELR, EMR, and ENR were higher in children than in adults (Tables 1 and 2). The mean ELR, EMR, and ENR of adults were 0.09, 0.45, and 0.05, respectively, within 30 days of diagnosis. The mean ELR, EMR, and ENR of adults 12–18 months before diagnosis were 0.10, 0.44, and 0.05, respectively. Children’s mean ELR, EMR, and ENR within 30 days of diagnosis were 0.10, 0.60, and 0.11, respectively. The mean ELR, EMR, and ENR of children 12–18 months before the diagnosis were 0.09, 0.52, and 0.10, respectively.

**Table 2:** Hematologic ratios of patients with chronic vitligo, stratified by age group.

	Adults	SD	Children	SD	p-value
n	8398		1428		
**EMR (mean (SD))**	**0.45**	**0.41**	**0.60**	**0.59**	**<0.001**
ELR 12–18 months before (mean (SD))	0.10	0.08	0.09	0.09	0.49
**ENR 12–18 months before (mean (SD))**	**0.05**	**0.05**	**0.10**	**0.13**	**<0.001**
**EMR 12–18 months before (mean (SD))**	**0.44**	**0.39**	**0.52**	**0.51**	**0.002**

SD: Standard Deviation

ELR: Eosinophil Lymphocyte Ratio

ENR: Eosinophil Neutrophil Ratio

EMR: Eosinophil Monocyte Rati

P-value < 0.05 was considered statistically significant

*T-tests were used for all variables

8,951 controls matched for age and gender were compared to patients with vitiligo (Table 3). Patients had a slightly higher EMR and ENR than their controls (0.47 vs 0.45, p = 0.02, and 0.06 vs 0.05, p<0.001, respectively). However, there was no significant difference in ELR between patients with vitiligo and controls.

**Table 3:** Hematological parameters of all patients with vitiligo compared to healthy controls.

	Controls	SD	Patients	SD	p-value
n	8951		8951		
ELR (mean (SD))	0.09	0.08	0.09	0.08	0.606
**ENR (mean (SD))**	**0.05**	**0.06**	**0.06**	**0.06**	**<0.001**
**EMR (mean (SD))**	**0.45**	**0.42**	**0.47**	**0.42**	**0.02**

SD: Standard Deviation

ELR: Eosinophil Lymphocyte Ratio

ENR: Eosinophil Neutrophil Ratio

EMR: Eosinophil Monocyte Ratio

P-value < 0.05 was considered statistically significant

*T-tests were used for all variables

Patients with mild vitiligo were compared to their matched controls in Table 4. There was a statistical difference in ENR between these populations. Mild patients had a higher ENR than their matched controls (0.06 vs 0.05, p<0.001). There was no significant difference in ELR or EMR.

**Table 4:** Hematological parameters of mild vitiligo patients compared to healthy controls.

	Controls	SD	Mild Patients	SD	p-value
n	7992		7992		
ELR (mean (SD))	0.09	0.08	0.09	0.08	0.205
**ENR (mean (SD))**	**0.05**	**0.05**	**0.06**	**0.06**	**<0.001**
EMR (mean (SD))	0.45	0.41	0.46	0.42	0.174

SD: Standard Deviation

ELR: Eosinophil Lymphocyte Ratio

ENR: Eosinophil Neutrophil Ratio

EMR: Eosinophil Monocyte Ratio

P-value < 0.05 was considered statistically significant

*T-tests were used for all variables

Patients with severe vitiligo were compared to their matched controls in Table 5. There was a statistically significant difference in EMR and ENR between the two groups. Severe vitiligo patients had a higher EMR (0.50 vs. 0.44, p = 0.003) and higher ENR (0.06 vs. 0.05, p = 0.001), compared with their matched control.

**Table 5:** Clinical, demographic, and laboratory characteristics of severe vitiligo patients compared to healthy controls.

	Controls	SD	Severe Patients	SD	p-value
n	959		959		
ELR (mean (SD))	0.10	0.10	0.10	0.08	0.07
**ENR (mean (SD))**	**0.05**	**0.07**	**0.06**	**0.06**	**0.001**
**EMR (mean (SD))**	**0.44**	**0.44**	**0.50**	**0.45**	**0.003**

SD: Standard Deviation

ELR: Eosinophil Lymphocyte Ratio

ENR: Eosinophil Neutrophil Ratio

EMR: Eosinophil Monocyte Ratio

P-value < 0.05 was considered statistically significant

*T-tests were used for all variables

**Fig 2:**
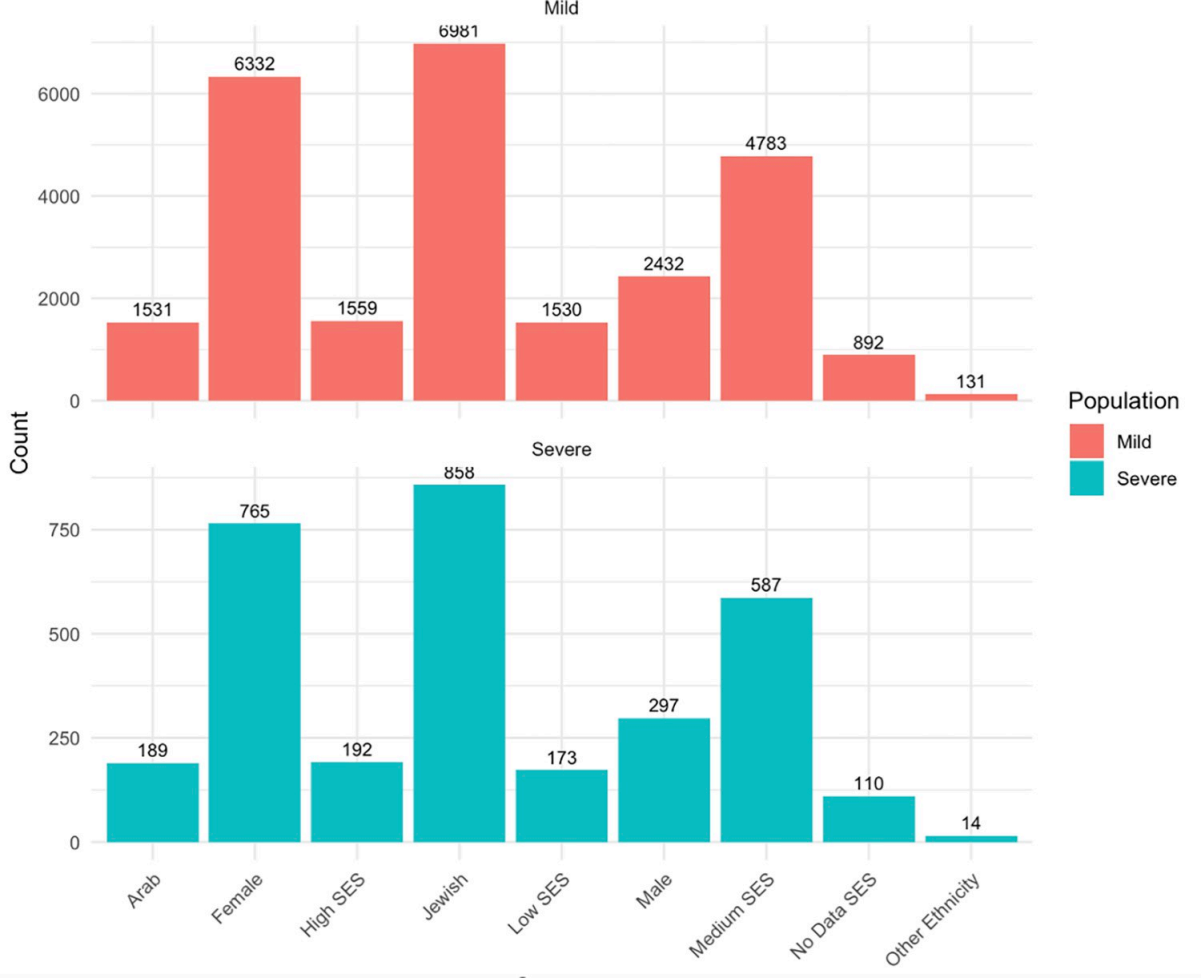
Demographics of patients with mild versus severe vitiligo.

Patients with mild vitiligo were compared to patients with severe vitiligo in Tables 6 and 7 and Fig 2. One thousand and sixty-two (28.0%) patients were defined as having a severe case of vitiligo. Severe vitiligo patients were significantly older than patients with mild vitiligo (43.98 years vs. 39.90 years, p<0.001). There was no significant difference in gender, smoke status, or socioeconomic score between patients with mild and severe vitiligo.

**Table 6:** Demographic characteristics of patients with vitiligo, stratified by severity group.

	Mild	SD	Severe	SD	p-value
n	8764		1062		
**Gender (%)**					
Male	2432 (27.7)		297 (28.0)		0.91
Female	6332 (72.3)		765 (72.0)		
**Age (mean (SD))**	**39.90**	**21.16**	**43.98**	**22.71**	**<0.001**
Socioeconomic score (%)					0.82
High	1559 (17.8)		192 (18.1)		
Medium	4783 (54.6)		587 (55.3)		
Low	1530 (17.5)		173 (16.3)		
No Data	892 (10.2)		110 (10.4)		
**Ethnicity (%)**					**0.01**
**Arab**	**1531 (17.5)**		**189 (17.8)**		
**Jewish**	**6981 (79.7)**		**858 (80.8)**		
**Other**	**131 (1.5)**		**14 (1.3)**		

SD: Standard Deviation

P-value < 0.05 was considered statistically significant

*Chi square test was used for all variables

**Table 7:** Laboratory characteristics of patients with vitiligo, stratified by severity group.

	Mild Vitiligo Patients	SD	Severe Vitiligo Patients	SD	P-value
**ELR (mean (SD))**	**0.09**	**0.09**	**0.10**	**0.09**	**<0.001**
**ENR (mean (SD))**	**0.06**	**0.07**	**0.07**	**0.08**	**<0.001**
**EMR (mean (SD))**	**0.47**	**0.44**	**0.52**	**0.50**	**<0.001**
**ELR 12–18 months before (mean (SD))**	**0.09**	**0.08**	**0.10**	**0.08**	**0.02**
**ENR 12–18 months before (mean (SD))**	**0.05**	**0.06**	**0.06**	**0.06**	**0.01**
**EMR 12–18 months before (mean (SD))**	**0.44**	**0.39**	**0.49**	**0.43**	**0.01**

SD: Standard Deviation

ELR: Eosinophil Lymphocyte Ratio

ENR: Eosinophil Neutrophil Ratio

EMR: Eosinophil Monocyte Ratio

P-value < 0.05 was considered statistically significant

*T-tests were used for each variable

Compared to mild vitiligo patients, patients with severe vitiligo had a higher ELR, EMR, and ENR both at the time of diagnosis and 12–18 months before the diagnosis. ELR, EMR, and ENR of patients with severe vitiligo were 0.10, 0.52, and 0.07, respectively. ELR, EMR, and ENR of patients with mild vitiligo were 0.09, 0.47, and 0.06, respectively (p<0.001). ELR, EMR, and ENR 12–18 months before vitiligo diagnosis of severe patients compared to mild patients were 0.10 vs. 0.09 (p = 0.02), 0.49 vs. 0.44 (p = 0.01), and 0.06 vs. 0.05 (p = 0.01), respectively.

Through multivariable analysis (Table 7), we observed that patients with higher ENR, EMR, and ELR at the time of diagnosis, ENR 12–18 months before the diagnosis, and EMR 12–18 months before diagnosis had higher odds of having severe vitiligo (OR = 7.40, p<0.001, OR = 1.27, p<0.001, OR = 3.17, p<0.001, OR = 7.19, p = 0.003, and OR = 1.29, p = 0.010, respectively).

Youden indexes displayed in Table 8 identified the best cut-off values for predicting severe vitiligo. Cut-off values of 0.09, 0.35, and 0.09 were optimal for ELR, EMR, and ENR at the time of diagnosis (AUCs = 0.55, 0.54, and 0.55, respectively). Cut-offs of 0.51 and 0.06 for EMR and ENR 12–18 months before the diagnosis of vitiligo were best at predicting severe vitiligo (AUCs = 0.54).

**Table 8:** Regression for severe vitiligo.

Characteristic7	OR^1^	95% CI^2^	p-value
**ENR**	**7.40**	**3.32, 16.2**	**<0.001**
**EMR**	**1.27**	**1.12, 1.44**	**<0.001**
**ELR**	**3.17**	**1.69, 5.87**	**<0.001**
**ENR 12–18**	**7.19**	**1.87, 26.7**	**0.003**
**EMR 12–18**	**1.29**	**1.06, 1.56**	**0.010**
ELR 12–18	2.27	0.86, 5.70	0.087

^1^ OR = Odds Ratio

^2^CI = Confidence Interval

P-value < 0.05 was considered statistically significant

## Discussion

We found significant differences in blood count parameters between adults and children, severe and mild vitiligo patients, and vitiligo patients with their matched controls. We found that eosinophil hematologic ratios were increased in vitiligo patients compared to controls and patients with severe vitiligo compared to mild vitiligo. We observed that patients with higher ELR, ENR, and EMR at the time of diagnosis and ENR and EMR 12–18 months before diagnosis of vitiligo had a significantly higher odds of having severe vitiligo.

Children with vitiligo had a higher ELR, EMR, and ENR than adults. This may reflect the underlying decrease in neutrophils and increase in lymphocytes, eosinophils, and platelets in children. Previously published studies have also demonstrated a significant increase in the median peak eosinophil count in children compared to adults with hypereosinophilic syndrome, possibly indicating an eosinophilic response of increased proportion in children compared to adults [7]. This enhanced response in children may be responsible for our findings in eosinophil counts and increased ELR, EMR, and ENR in children.

Patients with vitiligo had a higher EMR and ENR when compared to their controls matched for age and gender. There was no significant difference in ELR between patients with vitiligo and controls. Our findings follow a general trend of increased ENR and EMR with disease presence.

Notably, patients with vitiligo had higher EMR and ENR compared to their matched controls. Though EMR and ENR have both been associated with poor outcome and mortality in other diseases, there have been no previous studies investigating EMR and ENR in vitiligo compared to controls [8]. Eosinophils have been shown to play a role in several autoimmune conditions including autoimmune myocarditis, inflammatory bowel diseases, neuromyelitis optica, bullous pemphigoid, primary biliary cirrhosis, eosinophilic granulomatosis with polyangiitis among others [6]. Eosinophils are known for their cytotoxic abilities mediated primarily by granule release. Other proposed mechanisms of eosinophil-mediated tissue destruction include degranulation of cytotoxic proteins, induction of antibody-dependent cell-mediated cytotoxicity, release of proteases and cytokines, and enhancement of antigen detection and thrombotic function [6]. A relative increase in eosinophils compared to neutrophils and monocytes in vitiligo patients may contribute to our findings and highlight an essential role of eosinophils in vitiligo.

Severe vitiligo patients had higher ELR, EMR and ENR than mild vitiligo patients both at the time of diagnosis and 12–18 months before the diagnosis of vitiligo. ELR has been associated with systemic inflammatory activity and increased morbidity and mortality. In skin disease, ELR has been correlated with degree of itch in atopic dermatitis [8]. Higher ELR has also been associated with severe chronic spontaneous urticaria [9]. In addition, EMR and ENR have both been associated with poor outcomes and mortality in acute ischemic stroke patients and chronic obstructive pulmonary disease patients [10, 11]. Though the underlying mechanism is still unknown, it is possible that a relative increase in eosinophils plays a role in not only the pathogenesis of vitiligo, but also in the severity of vitiligo.

Our study further indicated that patients with an increase in ELR, ENR, and EMR at the time of diagnosis and ENR and EMR 12–18 months before the time of diagnosis have higher odds of having severe vitiligo and receiving systemic medication. These findings shed light on the efficacy of these ratios in identifying severe vitiligo patients.

Although there have been previous publications investigating NLR in vitiligo patients, no previous studies have investigated eosinophil hematologic ratios in vitiligo patients. Our study has a few limitations. First, our population excluded patients with infection, history of surgery, and malignancy before the time of the blood test, however, we may have included patients with other conditions that affect blood count values. Secondly, we took data from 30 days before and after diagnosis, however, it is uncertain whether the disease was present at that time or had not yet begun. Finally, due to the retrospective nature of this study, we were unable to determine vitiligo severity by area (Vitiligo Area Scoring Index) or subdivide stable/active and segmental/generalized subtypes of vitiligo. The severe population may have differences in blood counts due to the systemic medication they were prescribed. Nevertheless, we are the first study, to our knowledge, to compare eosinophils ratios in a large semi-national population between children and adults, patients and controls, and mild and severe vitiligo patients.

## Conclusion

As demonstrated in our study, there is a role for hematological markers, specifically eosinophil ratios, in the assessment of vitiligo presence, severity, and prognosis. We determined elevated Eosinophil to Lymphocyte Ratio, Eosinophil to Neutrophil Ratio, Eosinophil to Monocyte Ratio are associated severe vitiligo. The exact pathogenesis and mechanism behind the role of eosinophils, monocytes, and neutrophils in vitiligo need to be further investigated.

## Supporting information

S1 Checklist STROBE statement—checklist of items that should be included in reports of observational studies. (DOC)

S1 Table Youden indexes for severe vitiligo. (DOCX)
