# Supplementary material for: Determination of elevated eosinophil to lymphocyte ratio, eosinophil to neutrophil ratio, eosinophil to monocyte ratio and its association with severe vitiligo: A retrospective cohort study
Source: PLoS One. 2024 Feb 12;19(2):e0296626. doi: 10.1371/journal.pone.0296626 (PMC10861031; doi:10.1371/journal.pone.0296626)
Supplement: S1 Table — (DOCX) [file pone.0296626.s002.docx]

Supplementary Table 1. Youden Indexes for severe vitiligo

| Characteristic | | Threshold | | Specificity | | Sensitivity | | AUCs | |
| --- | --- | --- | --- | --- | --- | --- | --- | --- | --- |
| **ELR** | | | **0.09** | | **0.64** | | **0.44** | | **0.55** |
| **EMR** | | | **0.35** | | **0.51** | | **0.56** | | **0.54** |
| **ENR** | | | **0.09** | | **0.64** | | **0.44** | | **0.55** |
| ELR 12-18 | | | 0.06 | | 0.42 | | 0.66 | | 0.54 |
| **EMR 12-18** | | | **0.51** | | **0.72** | | **0.36** | | **0.54** |
| **ENR 12-18** | | | **0.06** | | **0.42** | | **0.66** | | **0.54** |
|  | ^1^ AUC = Area Under the Curve | | | | | | | | |
